# Codon Usage Bias of the Polyphenol Oxidase Genes in *Camellia sinensis*: A Comprehensive Analysis

**DOI:** 10.3390/plants14193074

**Published:** 2025-10-04

**Authors:** Yeşim Aktürk Dizman

**Affiliations:** Department of Biology, Faculty of Arts and Sciences, Recep Tayyip Erdoğan University, 53100 Rize, Türkiye; yesim.akturk@erdogan.edu.tr

**Keywords:** *Camellia sinensis*, polyphenol oxidase, codon usage bias, natural selection, mutation pressure

## Abstract

Tea, a widely consumed beverage globally, is a vital agricultural product for many countries. Polyphenol oxidases (PPOs), copper-containing enzymes found in plants, fungi, and animals, are essential for physiological metabolism and enzymatic browning in tea plants (*Camellia sinensis*). Codon usage bias (CUB), a key evolutionary characteristic, offers valuable insights into species evolution and gene function. However, the codon usage patterns of *Camellia sinensis* polyphenol oxidase (*CsPPO*) genes remain undocumented. In this study, we conducted, for the first time, a comprehensive analysis of CUB in 24 *CsPPO* genes, comparing their CUB profiles with those of other *Camellia* species (*Camellia lanceoleosa*, *Camellia nitidissima*, *Camellia ptilophylla*) and non-*Camellia* species (*Actinidia chinensis*, *Cornus florida*, *Rhododendron vialii*) to elucidate potential evolutionary relationships and functional constraints influencing CUB. Nucleotide composition analysis revealed an AT-rich bias, with a preference for G/C-ending codons at the third position. Codon usage indices indicated low expression levels and weak CUB. RSCU and RFSC analyses revealed that the preferred and high-frequency codons were mostly G/C-ending. Codon usage frequency analysis suggested *Zea mays* as a suitable host for *CsPPO* gene expression. ENC-GC3s, PR2, and neutrality plots showed natural selection had a stronger impact than mutation on CUB. Additionally, measure independent of length and composition (MILC) values confirmed low *PPO* gene expression levels, and correlation analyses demonstrated that both nucleotide composition and gene expression affect CUB. Overall, codon usage in *CsPPO* genes is mainly shaped by natural selection, with weak bias and low expression potential, providing useful insights for future genetic engineering and heterologous expression.

## 1. Introduction

*Camellia sinensis*, widely recognized as the tea plant, is a perennial evergreen woody species cultivated across a broad geographical range extending from tropical to temperate climates. *C. sinensis*, a member of the Theaceae family, is a crop of significant commercial and medicinal value, primarily due to its rich secondary metabolites and its widespread popularity as an aromatic beverage with a distinctive flavor. The leaves of *C. sinensis* are rich in various secondary metabolites, including polyphenols, vitamins, alkaloids, volatile oils, and polysaccharides [1,2,3]. Tea polyphenols are primarily composed of catechins, phenolic acids, and flavanones [4]. Polyphenols make up about 36% of the dry weight of young tea leaves, playing crucial roles not only in plant physiology but also in promoting human health [5]. Polyphenol oxidase (PPO), a key metalloenzyme in *C. sinensis*, is encoded and expressed by nuclear genes [6]. PPO, a vital enzyme in tea production, catalyzes the transformation of key phenolic metabolites into various derivatives, thereby influencing the extent of tea oxidation and significantly shaping its flavor, color, and taste [7]. Apart from its involvement in tea processing, PPO is also vital for stress responses, plant defense, and the regulation of secondary metabolism [8,9]. Due to the functional significance of *PPO* genes in tea plants, elucidating their genetic and evolutionary features is crucial. Codon usage bias, a key determinant of gene expression and evolutionary dynamics, offers valuable insights into the regulatory mechanisms and adaptive evolution of *PPO* genes. Accordingly, clarifying codon usage in *CsPPO* genes can provide insights into how translational constraints have shaped oxidative metabolism and stress response capacity in tea plants, two key biological attributes that underpin both tea quality and environmental adaptation in *C. sinensis*.

Codons play a fundamental role in the transmission of genetic information, serving as the critical link between nucleotide sequences and their corresponding amino acids during protein synthesis in living organisms. Amino acids may be encoded by between one and six different codons, a characteristic known as codon degeneracy [10]. Among these, codons that correspond to the same amino acid are referred to as synonymous codons, and they are not used with equal frequency during translation [11]. The preferential utilization of specific synonymous codons over others is termed codon usage bias (CUB). Previous studies have revealed that multiple factors influence CUB patterns, such as natural selection, gene length, mutation pressure, genome organization, levels of gene expression, and the structure of the tRNA anticodon-binding domain [12,13]. So far, studies have shown that the frequency of synonymous codon usage varies unevenly among different species, between nuclear and organelle genes, and even within individual genes [14,15]. Investigating CUB in specific genes or genomes is essential for understanding the molecular mechanisms of gene expression and for designing efficient expression vectors to enhance the production of target genes [12]. Optimal codon usage reflects molecular strategies organisms employ to adapt to their environments, thereby revealing the evolutionary forces that shape long-term genomic evolution [16]. Consequently, analyzing CUB provides valuable insights into both the evolutionary trajectories and expression patterns of genes. Consistent with this view, in *C. sinensis*, examining the balance between selection and mutation in the codon usage of *CsPPO* genes provides a valuable framework for understanding how the tea plant has evolutionarily optimized PPO activity to regulate phenolic metabolism while simultaneously maintaining resilience under both biotic and abiotic stresses.

CUB has been widely studied in several plant species, including *Helianthus annuus* [13], *Gynostemma* species [17], *Populus* species [15], and *Coffea* species [18], contributing to our understanding of molecular biology, genetics, and the evolutionary dynamics of plant genomes [19]. In tea plants, *PPO* genes are of particular interest because they play crucial roles in physiological metabolism and enzymatic browning. Although numerous studies have investigated the characteristics and phylogenetic relationships of nuclear genes in *C. sinensis* [20,21], the codon usage patterns of *PPO* genes in this species remain unexplored. The objective of this study was to systematically analyze codon usage bias in 24 *CsPPO* genes, identify the factors influencing their codon usage, and compare these patterns with *PPO* genes from other *Camellia* species (*C. lanceoleosa*, *C. nitidissima*, *C. ptilophylla*) and non-*Camellia* species (*A. chinensis*, *C. florida*, *R. vialii*). Using nucleotide composition analysis, codon usage indices, and correlation approaches, this work represents the first comprehensive investigation of *CsPPO* codon usage patterns. The significance of this study lies in its potential applications for optimizing *PPO* gene expression in synthetic biology, genetic engineering, and crop improvement programs. Identifying optimal codons in *PPO* genes can guide the development of stable transgenic systems, facilitate efficient heterologous expression, and enable targeted modifications in metabolic pathways to improve tea quality and stress resilience. Ultimately, this research provides a broader perspective on plant gene evolution and the molecular mechanisms regulating secondary metabolism in tea plants.

## 2. Results

### 2.1. Patterns of Codon Composition

A key consideration is that genomic nucleotide composition can significantly shape CUB [22]. This study examined the nucleotide composition of the 24 *CsPPO* genes to assess its potential influence on CUB (Table 1 and Table S1). The nucleotide frequencies of *CsPPO* genes were as follows: A (27.87%), T (23.27%), C (24.15%), and G (24.71%). To further evaluate whether this nucleotide composition pattern is species-specific, we performed a comparative analysis with *PPO* genes from a diverse set of plant species. Specifically, we included *PPO* genes from other *Camellia* species as well as non-*Camellia* species representing broader taxonomic diversity. This comparative analysis revealed that the average nucleotide composition across these *PPO* genes was A (27.09%), T (23.80%), C (24.40%), and G (24.71%) (Table 1 and Table S1). These values demonstrate a modestly higher A content in *CsPPO* genes, possibly indicating species-specific compositional features. Nucleotide composition at the third codon position was also analyzed. *CsPPO* genes exhibited A3s (28.38%), T3s (31.25%), G3s (35.04%), and C3s (33.38%), while the average values across other *PPO* genes were A3s (26.26%), T3s (34.16%), G3s (33.88%), and C3s (32.56%). These differences suggest that *CsPPO* genes exhibit a stronger preference for G and C at the third codon position, and a reduced usage of A/T, relative to other species. The mean AT and GC content in *CsPPO* genes was 51.13% and 48.87%, respectively, which is comparable to the mean AT (50.89%) and GC (49.11%) contents observed in other *PPO* genes. AT3 values in *CsPPO* genes ranged between 45.00% and 55.26%, with a mean of 46.25%, which is slightly lower than the AT3 average of 47.27% in other *PPO* sequences. The GC content at various codon positions serves as a critical indicator of nucleotide composition bias. Meanwhile, the GC3s content in *CsPPO* genes was 52.50%, slightly higher than the 51.31% average of other *PPO* genes, suggesting a moderate preference for G/C-ending codons in *CsPPO* genes. Furthermore, GC content at the first and second codon positions in *CsPPO* genes was 52.70% and 40.15%, respectively, compared to 52.76% (GC1) and 41.84% (GC2) in *PPO* genes from other species. These values indicate that while GC1 content is relatively conserved, GC2 content is somewhat reduced in *CsPPO* genes. These nucleotide composition patterns, particularly at the third codon position, may contribute to CUB and could reflect species-specific evolutionary pressures or functional adaptations in *C. sinensis*.

### 2.2. Codon Usage Indices Analysis

In this study, ENC values of *CsPPO* genes varied between 49.01 and 57.73, with a mean of 56.34 (Table 2 and Table S1), indicating relatively low codon usage bias. To determine whether the codon usage indices of *CsPPO* genes reflect species-specific trends, we compared them with *PPO* genes from other *Camellia* species and non-*Camellia* species (Table 2 and Table S1). The average ENC value across these species was 56.21, which is slightly lower than that of the *CsPPO* genes, suggesting that codon usage in *CsPPO* genes may be slightly less constrained. The CAI values of *CsPPO* genes exhibited a range of 0.162–0.204, with a mean value of 0.195. Given the generally positive correlation between codon adaptation index and gene expression levels, these CAI values suggest that *CsPPO* genes are likely to be expressed at relatively low levels. The mean CAI value of *PPO* genes from other species was similar (0.196), indicating comparable levels of translational adaptation. The CBI values of *CsPPO* genes spanning –0.239 to –0.015, averaging –0.058, suggesting a largely random pattern in the use of non-preferred codons among these genes. Other plant *PPO* genes exhibited a similar average CBI of –0.065, further supporting the absence of strong codon bias across these genes. GRAVY and AROMA metrics were also examined to characterize the physicochemical properties of the PPO proteins. *CsPPO* genes had a mean GRAVY value of –0.476, supporting their hydrophilic nature, while other *PPO* genes showed a comparable mean of –0.464. The AROMO values in *CsPPO* genes ranged from 0.046 to 0.093, with a mean of 0.090, closely matching the 0.091 average observed in other plants *PPO* genes. These results indicate that the aromatic amino acid composition is generally conserved across *PPO* genes regardless of species origin.

### 2.3. RSCU and RFSC Analyses and Determination of High-Frequency Codons

CUB was assessed through RSCU and RFSC analyses. The results revealed that 26 codons (RSCU > 1) were preferred in the *CsPPO* genes (Table S2). Of these, 16 codons had G/C endings (9 ending in C and 7 in G), while the remaining ten had A/T endings. These results provided evidence that the codons of *CsPPO* genes predominantly ended in G/C, indicating a bias in synonymous codon usage influenced by compositional constraints. Additionally, four codons (CTC, GTG, ACC, CGA) were classified as over-represented (RSCU > 1.6), and eight (CTA, CTG, ATC, GTT, GTA, ACG, GCA, CGC) as under-represented (RSCU < 0.6), indicating differential selection pressures among synonymous codons in the *CsPPO* genes. To further assess whether codon usage trends in *CsPPO* genes are species-specific, we conducted a comparative RSCU and RFSC analyses using *PPO* genes from other *Camellia* species and non-*Camellia* species (Table S2). In *C. lanceoleosa*, 29 preferred codons were detected, with 14 ending in G/C and 10 ending in A/T. Two codons (GTG, ACC) were over-represented, while eight were under-represented (GTT, TCG, ACG, CAC, GAC, CGT, CGC, GGC). In *C. nitidissima*, 33 preferred codons were observed, including 17 ending in G/C and 10 in A/T. Four codons (TTG, GTG, ACC, AGA) were over-represented, whereas eight (CTG, GTT, GTA, ACG, GAC, CGT, CGC, GGC) were under-represented. In *C. ptilophylla*, 27 preferred codons were identified, with 15 ending in G/C and 10 in A/T. Three codons (CTC, GTG, CGA) were over-represented, while nine (CTG, ATC, GTT, GTA, TCA, ACG, GCA, TGT, CGC) were under-represented. Among the non-*Camellia* species, *A. chinensis* exhibited 30 preferred codons, of which 19 ended in G/C and 11 in A/T. Two codons (GTG, AGG) were over-represented, and seven (GTT, GTA, AGT, ACA, GAA, CGC, CGA) were under-represented. In *C. florida*, 30 preferred codons were detected, with 12 ending in G/C and 18 in A/T. Four codons (TTG, GTG, GCT, AGG) were over-represented, whereas eleven (TTA, GTC, GTA, ACG, GCA, CAC, CAG, CGC, CGA, CGG, GGC) were under-represented. In *R. vialii*, 28 preferred codons were identified, including 18 ending in G/C and 10 in A/T. Seven codons (ATC, GTG, TCC, ACC, GCC, AGA, AGC) were over-represented, while eleven (CTG, ATA, GTA, TCA, CCT, ACG, CAG, CGT, CGC, CGA, GGC) were under-represented. Overall, the data demonstrate that *PPO* genes in *Camellia* species generally exhibit a preference for G/C-ending codons, although the extent of over- and under-representation of specific codons varies among species, reflecting both compositional constraints and species-specific selection pressures.

By calculating the RFSC values of both *CsPPO* genes and *PPO* genes from other plants, high-frequency codons were identified, and in line with the approach of Zhou et al. [23] those with a relative frequency greater than 60% (the proportion of a given codon among the total synonymous codons for a particular amino acid) were classified as high-frequency codons. The high-frequency codons in the *CsPPO* genes are listed in Table 3 and Table S2. A total of 18 high-frequency codons were identified, including TTC, CTC, ATT, GTG, TCC, CCG, ACC, GCC, TAC, CAT, CAA, AAC, AAG, GAT, GAG, TGC, CGA, and GGG. It was noted that the majority of these high-frequency codons preferentially ended in G/C. However, the ACG codon in the *CsPPO* genes exhibited a low RSCU value, which could help mitigate potential mutations associated with DNA methylation [24]. The GTA codon in *CsPPO* genes exhibited relatively low RSCU values, and the decrease in TA could enhance protein synthesis by preventing mRNA degradation [25]. In the *PPO* genes of other plant species, 20 high-frequency codons were identified in *C. lanceoleosa*, 21 in *C. nitidissima*, 19 in *C. ptilophylla*, 17 in *A. chinensis*, 20 in *C. florida*, and 20 in *R. vialii* (Table 3 and Table S2) Comparative analysis revealed seven high-frequency codons (GTG, TCC, ACC, CAA, AAG, GAT, GAG) shared between the *CsPPO* genes and the *PPO* genes of these species. Overall, high-frequency codon profiles demonstrated a general bias toward G/C-ending codons across all species, although the specific codon composition varied, indicating both conserved and species-specific codon usage preferences.

### 2.4. Determination of Optimal Codons

Gene datasets representing high and low expression levels were established based on the ENC values of each *PPO* coding sequence. Subsequently, the RSCU and ΔRSCU values were computed using the CodonW. Optimal codons were identified based on ΔRSCU values exceeding 0.08, with RSCU values greater than 1 in high-bias genes (high expressed genes) and less than 1 in low-bias genes (low expressed genes) (Table S3) [13,26]. The analysis revealed that the *CsPPO* genes included eight optimal codons: TTT, ATA, CCT, GCT, AAT, CGG, AGG, and GGT. Likewise, optimal codon analysis of *PPO* genes from other *Camellia* species and non-*Camellia* plant species identified a total of 13 optimal codons: TTT, CTT, ATA, GTT, TCA, CCT, ACA, GCT, TAT, AAT, TGT, AGG, and GGT. The findings revealed that seven optimal codons in the *PPO* genes (TTT, ATA, CCT, GCT, AAT, AGG, and GGT) were shared between *C. sinensis*, other *Camellia* species, and non-*Camellia* plant species.

### 2.5. Codon Usage Frequency Analysis

Transgenic research frequently relies on heterologous gene expression, which can be influenced by numerous factors. Among these, the selection of optimal codons is one of the most critical determinants for successful expression of exogenous genes in the host. Differences in codon usage bias between the *CsPPO* genes and the host organisms can substantially affect gene expression levels; therefore, CUB must be carefully considered when investigating *CsPPO* gene expression in an exogenous system. In this study, the codon usage frequencies of the *CsPPO* genes and *PPO* genes from other plant species were analyzed and compared with those of six other species: *Escherichia coli*, *Saccharomyces cerevisiae*, *Arabidopsis thaliana*, *Nicotiana tabacum*, *Triticum aestivum*, and *Zea mays* (Table S4). The results revealed differences in codon usage frequencies between the *CsPPO* gene and the six model species, listed in decreasing order as follows: *Zea mays* (2–10 codons), *Triticum aestivum* (4–11), *Saccharomyces cerevisiae* (6–10), *Arabidopsis thaliana* (8–9), *Escherichia coli* (8–10), and *Nicotiana tabacum* (10–11). The codon frequency of the *CsPPO* gene showed only minor differences compared to that of *Zea mays*. In contrast, more pronounced differences were observed between the *CsPPO* gene and *Triticum aestivum*, *Saccharomyces cerevisiae*, *Arabidopsis thaliana*, *Escherichia coli*, and *Nicotiana tabacum*. In conclusion, the results indicate that *Zea mays* may be an optimal host for the heterologous expression of the *CsPPO* gene. Similarly, the results indicate that *Zea mays* may be an optimal host for the heterologous expression of the *PPO* genes of *C. lanceoleosa* (2–10 codons), *C. nitidissima* (3–9 codons), *C. ptilophylla* (2–12 codons), and *R. vialii* (2–8 codons). Furthermore, the results indicate that *Triticum aestivum* and *Arabidopsis thaliana* may be an optimal host for the heterologous expression of the *PPO* genes of *A. chinensis* (4–9 codons) and *C. florida* (3–7 codons), respectively. *Saccharomyces cerevisiae* and *Escherichia coli*, representing eukaryotic and prokaryotic expression systems, are commonly utilized in gene expression studies. In the present study, the *S. cerevisiae* expression system appears to be more favorable than the *Escherichia coli* system for the expression of the *CsPPO* gene.

### 2.6. ENC Plot Analysis of PPO Genes

**Figure 1:**
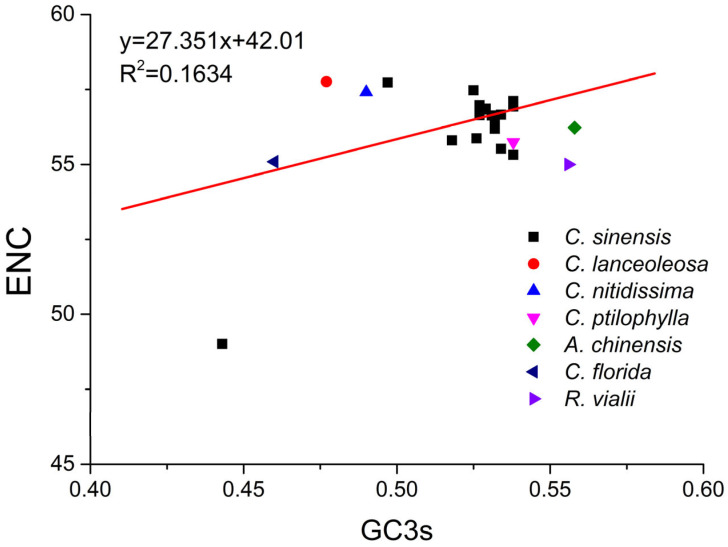
ENC-plot analysis of *PPO* genes. Each shape represents an individual gene, while the standard curve depicts the expected ENC values under the assumption of random codon usage.

The codon usage variation among the 24 *CsPPO* genes and *PPO* genes from other plant species was analyzed using an ENC plot (Figure 1). As depicted in Figure 1, a few genes clustered above or adjacent to the expected curve, implying mutational pressure impacted their codon usage. In contrast, most genes fell below the predicted curve, indicating that natural selection played the dominant role in shaping CUB.

### 2.7. PR2 Plot Analysis

**Figure 2:**
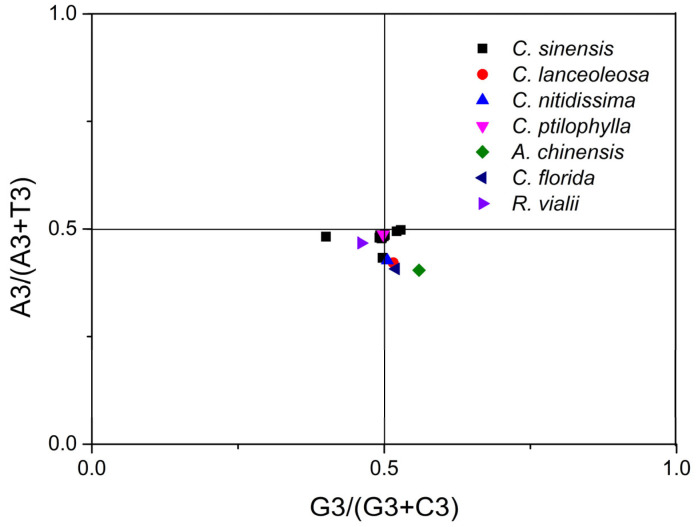
PR2 plot analysis between AT bias [A3/(A3 + T3] and GC bias [G3/(G3+ C3)] of *PPO* genes.

To distinguish the relative impacts of mutational pressure and natural selection on CUB, PR2 plot analysis was conducted (Figure 2). The analysis revealed that the 24 *CsPPO* genes, along with *PPO* genes from both *Camellia* and non-*Camellia* plant species, were unevenly distributed across the four regions, with most genes positioned far from the central value of 0.5. Only a few genes were located in proximity to the center. This finding implies that natural selection may exert a considerable influence on the usage patterns of the third codon base in these genes. Furthermore, the A3/(A3 + T3) ratio for the majority of codon bases was less than 0.5, whereas the G3/(G3 + C3) ratio surpassed 0.5 in certain gene codons. These results demonstrate that codon base usage, especially at the third position, tends to favor T over A and G over C.

### 2.8. Neutrality Plot Analysis

**Figure 3:**
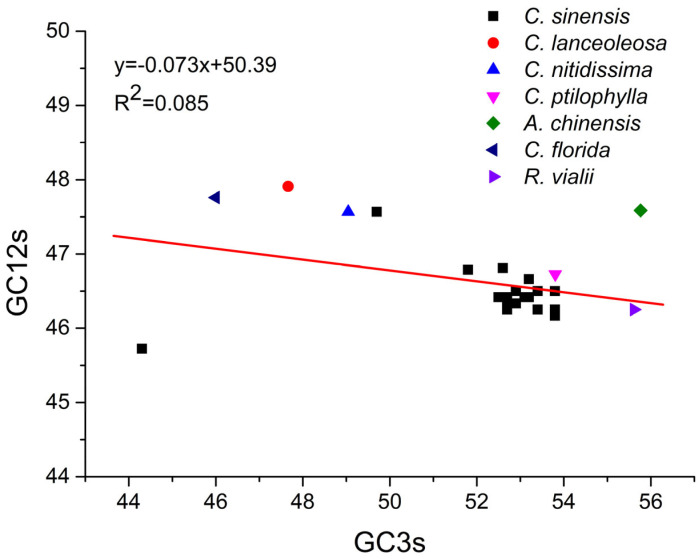
Neutrality plot analysis of *PPO* genes between GC12s and GC3s. The slope value reflects the proportion of mutational pressure contributing to the total variation.

The neutrality plot was employed to analyze the relationship between GC12s (the average GC content at the first and second codon positions) and GC3s, elucidating the respective roles of mutational pressure and natural selection in shaping codon usage patterns (Figure 3). Regression analysis based on 24 *CsPPO* genes and additional *PPO* genes from both *Camellia* and non-*Camellia* plant species yielded a slope of 0.073. The correlation coefficient (r = 0.291) indicated a weak association between GC12s and GC3s. This result suggests that mutational pressure contributed only 7.3% to the codon usage patterns of *PPO* genes, while other factors, primarily natural selection, accounted for the remaining 92.7%. In this context, natural selection plays a predominant role in shaping the codon usage patterns of both *CsPPO* genes and *PPO* genes from other plant species.

### 2.9. Correspondence Analysis of PPO Genes

**Figure 4:**
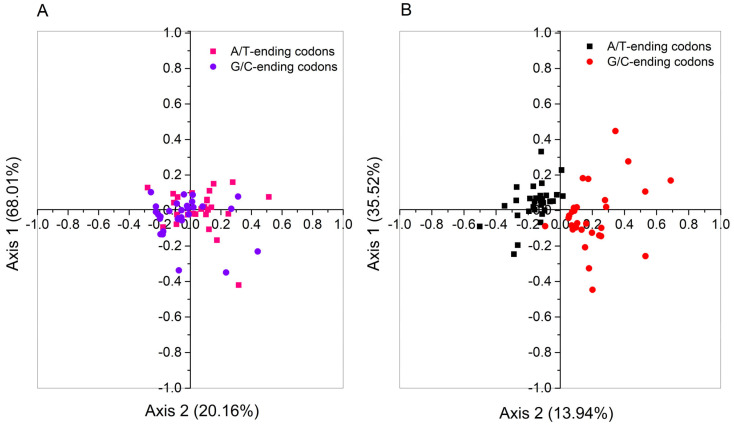
Correspondence analysis based on the RSCU values of *PPO* genes of *C. sinensis* (**A**) and other plant species (*Camellia lanceoleosa*, *Camellia nitidissima*, *Camellia ptilophylla*, *Actinidia chinensis*, *Cornus florida*, *Rhododendron vialii*) (**B**).

COA was conducted on codon usage patterns in *CsPPO* genes. The analysis was conducted using the RSCU values calculated from these genes. The results indicated that Axis 1 accounted for 68.01% of the total variation, whereas Axis 2 explained 20.16% of the overall variation (Figure 4A). AT-ending codons were predominantly localized near the central region of the plot, primarily within the positive quadrant, whereas GC-ending codons were mostly aggregated around the axis origin, with a few distributed toward the negative quadrant. To further examine codon usage divergence across species, an additional COA was performed using *PPO* genes from *Camellia* species and non-*Camellia* species. In this comparative analysis, Axis 1 and Axis 2 accounted for 35.52% and 13.94% of the total variation, respectively (Figure 4B). Codons were clearly separated along these axes based on their third nucleotide base: A/T-ending codons tended to cluster in specific regions distinct from those of G/C-ending codons. COA indicates that CUB in *PPO* genes results from a combination of mutational pressure, natural selection, and other potential contributing factors.

### 2.10. Amino Acid Usage Frequency

**Figure 5:**
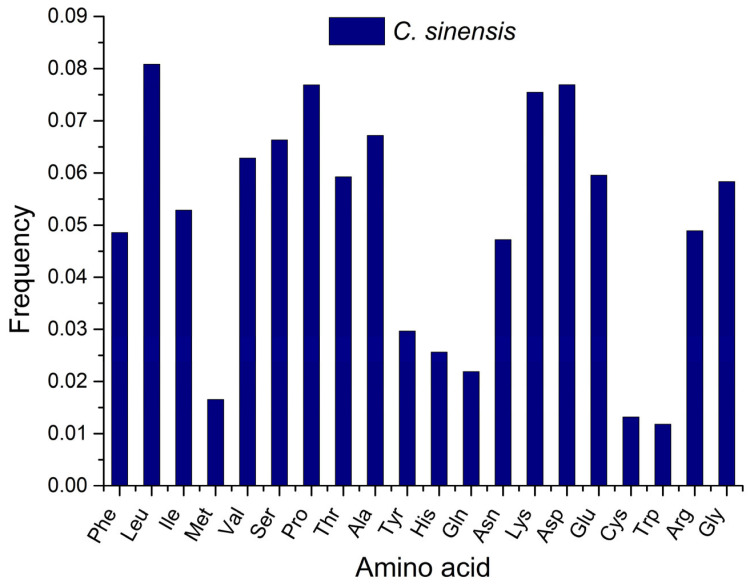
Amino acid usage frequencies analysis of *CsPPO* genes.

Amino acid composition acts as a vital indicator, revealing insights into an organism’s physiological functions, biological stability, and evolutionary trajectories [27]. Variations in genomic GC content have a direct effect on amino acid composition, subsequently affecting codon usage patterns [28]. In *CsPPO* genes, leucine, proline, aspartic acid, and lysine were the most abundant amino acids, whereas tryptophan, cysteine, and methionine were present at lower frequencies (Figure 5). Overall, hydrophobic and hydrophilic amino acids accounted for a substantial proportion of the complete amino acid profile in *CsPPO* genes. In addition to *CsPPO* genes, amino acid usage frequencies were also analyzed for *PPO* genes from other *Camellia* species and non-*Camellia* species (Table S5). The results showed that the most frequently used amino acids across all species were alanine, aspartic acid, leucine, proline, and lysine. In contrast, methionine, cysteine, and tryptophan were the least frequently used amino acids. These findings align with the patterns observed in *CsPPO* genes, in which hydrophobic and hydrophilic amino acids collectively constitute a major proportion of the overall amino acid composition.

### 2.11. Impact of Codon Usage Bias on Gene Expression

The MILC value serves as a measure of gene expression levels. Higher MILC values correspond to increased levels of gene expression [29]. We determined the MILC values for *CsPPO* genes and *PPO* genes of other plant species. The results indicated that these values ranged from 0.48 to 0.50 (Table 4), suggesting that the expression levels of *CsPPO* genes and the other plant species were low. Furthermore, we performed a correlation analysis to examine the association between gene expression levels and CUB. A significant positive correlation was found between SCUO and MILC values (Table S6), suggesting that CUB may influence gene expression levels.

### 2.12. Correlation Analysis

The association between nucleotide content and CUB indices was examined in the *CsPPO* genes. In *CsPPO* genes, ENC showed significant positive correlations with GC, GC2, GC3s, C3s, and A3s, while significant negative correlations were observed with GC1, T3s, G3s, and AT3 (Table 5). This indicates that codon usage bias could be associated with the nucleotide composition at different codon positions. Compared to GC1 and GC2, GC3 exerts a stronger influence on codon preference, particularly at the third position of synonymous codons.

While the composition of the first two bases is largely shaped by neutral mutations, the third base composition is predominantly governed by natural selection. Additionally, correlation analysis between CAI and various CUB indices showed that, in *CsPPO* genes, CAI is significantly positively correlated with GC1, T3s, and AT3s, while exhibiting a significant negative correlation with GC, GC2, GC3s, A3s, and C3s (Table 5). Gene expression levels, as assessed by CAI values, were found to influence CUB in the *CsPPO* genes. To assess natural selection’s influence on CUB in *CsPPO* genes, we examined correlations between GRAVY, AROMA and codon usage parameters (A3s, T3s, G3s, C3s, GC3s, ENC) (Table 6). The results showed significant associations between GRAVY, AROMA values and these parameters, suggesting that natural selection contributes to shaping CUB.

Furthermore, the association between nucleotide content and CUB indices was also analyzed in the *PPO* genes of other plant species (Table S7). In contrast to *CsPPO* genes, no significant correlation was detected between ENC and CAI values and any of the nucleotide composition parameters (GC, GC1, GC2, GC3s, A3s, T3s, C3s, G3s, AT3). However, a significant correlation was observed only between the ENC value and the GRAVY value in these species, which suggests that natural selection plays a substantial role in influencing codon usage patterns in these *PPO* genes, even in the absence of strong associations with nucleotide content.

## 3. Discussion

CUB is a complex and important evolutionary phenomenon observed across a wide range of organisms, providing valuable insights into genomic architecture and the evolutionary history of genomes [30]. CUB has been investigated across diverse species, encompassing prokaryotes as well as unicellular and multicellular eukaryotes [31,32]. Variation in synonymous codon usage across different gene-coding regions results from CUB and is shaped by selective forces, including nucleotide composition constraints, natural selection, and mutational pressure [33]. Studies on CUB have been conducted in various genes of *C. sinensis*, including *CsSAD*, *CsSPDS*, and *CsGPAT* [34,35,36]. However, this study presents the first comprehensive investigation of codon usage in *CsPPO* genes, comparing 24 *CsPPO* genes with *PPO* genes from other *Camellia* and non-*Camellia* species to assess codon usage patterns and evaluate the evolutionary forces shaping codon bias. This approach highlights the functional and evolutionary significance of *PPO* genes, which are directly linked to plant defense and stress responses, thereby extending codon usage studies to a biologically and agriculturally relevant gene family.

The nucleotide content and the 3rd codon position significantly influence the CUB of a gene [37]. In GC-rich organisms like *Triticum aestivum* [38], *Zea mays* [39], and *Sorghum bicolor* [40], there is a preference for G or C at the 3rd codon position. In contrast, AT-rich organisms, such as *Helianthus annuus* [13], *Delphinium grandiflorum* [41], and *Porphyra umbilicalis* [42], tend to favor A or T at the third codon position. In the present study on *PPO* genes of both *C. sinensis* and the other analyzed plant species, the average GC (G + C) content was found to be lower than the AT (A + T) content. However, at the third codon position, the mean GC3 content was higher than the AT3) content. Since GC3 composition is widely regarded as an indicator of base composition bias [43], our results suggest a bias toward the preferential use of G- or C-ending codons. These findings may reflect compositional constraints influencing the CUB of *CsPPO* genes and the *PPO* genes from other analyzed plant species. This observation was supported by the results of the RSCU analysis. Moreover, the RFSC and high-frequency codon analyses further confirmed a preference for GC-ending codons, aligning with trends reported in previous studies [44,45]. Earlier studies have suggested that the frequency of synonymous codon usage exhibits variation not only between genomes but also among functionally related genes and even within individual genes [46,47]. It is therefore widely accepted that variations in codon usage among genes within the same genome are largely driven by selective pressures, as evidenced by the stronger codon bias observed in highly expressed genes, which preferentially utilize codons corresponding to the most prevalent cognate tRNAs [48,49].

Selective pressures aimed at maximizing translational efficiency and accuracy play a central role in shaping codon usage patterns, as evidenced by the frequent use of optimal codons [50]. Identifying optimal codons offers valuable insights for rational and efficient codon optimization strategies [51,52,53]. In this study, eight optimal codons were identified in the *CsPPO* genes, and thirteen in the *PPO* genes of other analyzed plant species, respectively. These findings not only support efforts in codon optimization but also offer valuable insights into the association between gene expression and codon preference. Based on these results, our study emphasizes that codon modification and alignment with host genomes can significantly enhance transcriptional and translational efficiencies. This is particularly relevant for plant biotechnology, where optimized heterologous expression of PPO enzymes can contribute to improved stress tolerance, crop quality, and biotechnological production systems. In this context, our results highlight distinct host–gene compatibility patterns for *PPO* heterologous expression. *Zea mays* appears particularly well-suited for expressing the *CsPPO* gene and the *PPO* genes from *C. lanceoleosa*, *C. nitidissima*, *C. ptilophylla*, and *R. vialii*, suggesting a broad compatibility with phylogenetically diverse species. In contrast, *Triticum aestivum* and *Arabidopsis thaliana* emerged as optimal hosts for the *PPO* genes of *A. chinensis* and *C. florida*, respectively, indicating that host selection may be influenced by gene-specific or lineage-specific factors [54]. Taken together, these host–gene associations not only expand our understanding of codon bias but also provide a practical roadmap for designing effective plant-based expression systems in agriculture and synthetic biology.

CUB is mainly influenced by natural selection and mutation pressure [12], though the predominant forces driving this bias can differ between species. Previous studies have shown that the codon usage patterns of nuclear genes are largely influenced by natural selection throughout evolution [55,56,57]. In contrast, another study reported that mutation pressure primarily influences CUB in soybean nuclear genes [58]. These findings indicate that codon usage patterns of nuclear genes differ across plant species. A systematic analysis of the factors influencing CUB in the *CsPPO* genes and the *PPO* genes of other plant species was conducted using an integrated approach combining ENC plot, PR2 plot, and neutrality plot analyses. The ENC plot analysis provided a qualitative assessment of the principal determinants influencing codon usage patterns in *PPO* genes. The findings demonstrated that mutational bias exerted a relatively minor influence on codon usage patterns when compared to the effects of natural selection and other factors. Comparable findings have been reported in studies on *Rosales* species, *Hemerocallis citrina*, and *Citrus* species [59,60,61]. Neutrality plot analysis indicated the absence of a significant correlation between GC12 and GC3 in the *PPO* genes. In the *PPO* genes, mutation pressure contributed only 7.3% to the codon usage pattern, whereas natural selection accounted for 92.7%, suggesting that natural selection was the predominant force shaping codon preference. This result is consistent with previous studies on *Gnetales* species, *Diplandrorchis sinica*, and *Dryas octopetala* [62,63,64]. The PR2 plot analysis revealed an uneven distribution of *PPO* genes across the four quadrants, indicating a bias in the third codon position, with a higher usage frequency of T over A and G over C. These findings suggest that natural selection is the primary driver of codon usage bias in *PPO* genes. Comparable patterns have also been observed in other plants, including *Cymbidium* species and *Medicago truncatula* [65,66]. This strong predominance of selection underscores the adaptive nature of CUB and highlights its potential role in fine-tuning PPO expression under varying ecological and evolutionary contexts. However, to further investigate the factors shaping codon usage, COA of RSCU revealed that the first axis accounted for only a limited proportion of the codon usage variation, indicating that codon bias in the *CsPPO* genes and *PPO* genes from other plant species is shaped not solely by natural selection but also by additional selective constraints. This observation is reinforced by the distinct clustering pattern in the COA plot, which showed a clear separation between GC-ending and AT-ending codons, underscoring the role of nucleotide composition in shaping CUB [67]. Such a pattern is consistent with the influence of mutational pressure, where GC-rich genomes tend to favor G- or C-ending codons, whereas AT-rich genomes preferentially utilize A- or T-ending codons [68]. Furthermore, the potential contribution of translational selection—via differences in tRNA abundance or efficiency for GC- versus AT-ending codons—suggests that codon usage patterns are the outcome of an interplay between mutation bias and natural selection [50]. These results not only corroborate earlier findings that codon usage is a non-random phenomenon shaped by complex evolutionary forces [19,69], but also extend this understanding by demonstrating that such forces are at work in *PPO* genes across phylogenetically diverse plant species. This broader perspective highlights the evolutionary and functional significance of codon usage patterns in plant genomes and provides a framework for future studies on the molecular evolution of *PPO* genes.

The amino acid composition of proteins plays a crucial role in determining their structural configuration, functional performance, and evolutionary flexibility [28]. In the present study, both the *CsPPO* genes and the *PPO* genes of other plant species exhibited a distinctive amino acid composition, characterized by elevated abundances of leucine, proline, aspartic acid, and lysine, and reduced frequencies of tryptophan, cysteine, and methionine. The presence of both hydrophobic and hydrophilic amino acids in these *PPO* genes may indicate a carefully optimized balance between structural stability and enzymatic activity [70,71]. Hydrophobic amino acids like leucine and proline might aid in the proper folding and core stabilization of the PPO enzyme, which is critical for sustaining its activity under environmental stress [71,72,73]. Conversely, hydrophilic residues such as aspartic acid and lysine improve solubility and promote interactions with substrates and other biomolecules [74,75], thereby enhancing the enzyme’s function in oxidation reactions linked to defense responses and browning processes [76,77]. This structural–functional optimization suggests that selective forces act not only at the codon level but also at the amino acid level, reinforcing the adaptive nature of PPO evolution in response to biotic and abiotic pressures.

In our study, the SCUO values for the *PPO* genes in both *C. sinensis* and the other plant species were consistently below 0.50, indicating a low level of CUB and suggesting a relatively balanced use of synonymous codons. Such a pattern may reflect weaker selective constraints on codon choice, with mutational pressure or genetic drift playing a more prominent role in shaping codon usage. This observation is consistent with previous reports for chloroplast genes in *Oryza* and Theaceae species [78,79], thereby extending these findings to *PPO* genes and highlighting a potentially conserved codon usage strategy across phylogenetically distinct plant lineages. In our current study, MILC, together with ENC and CAI values, was employed as an indicator of gene expression and was found to exhibit low levels. These results suggest that, based on MILC, gene expression levels may vary to some extent among the different species. Based on gene expression levels, a correlation was observed between SCUO and MILC values, suggesting that CUB may influence gene expression. This trend aligns with previous findings in *Fagopyrum* species and Theaceae family members [29,79].

Previous studies have demonstrated that additional natural selection-driven factors, such as GRAVY and AROMO values, also contribute to CUB [80,81]. The strong positive correlations between GRAVY and AROMO values support the notion that these protein traits, shaped by the coding sequences, may be associated with CUB in *CsPPO* genes as well as in *PPO* genes from other plant species. Earlier studies have also identified notable positive and negative correlations between CUB and GRAVY/AROMO values in a range of organisms, such as *Sesamum indicum*, *Taenia saginata*, and *Epichloë festucae* [82,83,84]. Collectively, these observations highlight hydrophobicity and aromaticity as key selective forces shaping codon usage bias across plant species.

Despite these novel insights, certain limitations of our study should be acknowledged. Although chromosome-scale assemblies of *C. sinensis* exist, a fully comprehensive genomic resource covering the genetic diversity of tea is still lacking. This limits our ability to assess codon usage in a complete genomic context, where competition among mRNAs for tRNAs can be fully understood. Our results should therefore be seen as a first step, paving the way for broader genome-wide analyses. Future research that couples codon usage analyses with functional genomics and experimental validation (e.g., heterologous expression of *PPO* genes in different hosts) will be critical to clarify the adaptive significance of codon bias under natural and agricultural conditions. Such approaches will not only address current limitations but also extend the implications of our study to plant breeding, stress resilience, and synthetic biology applications.

## 4. Materials and Methods

### 4.1. Sequence Retrieval and Nucleotide Composition Analysis

In this study, *PPO* gene sequences from 24 *C. sinensis* isolates, other *Camellia* species (*Camellia lanceoleosa*, *Camellia nitidissima*, *Camellia ptilophylla*), and non-*Camellia* species (*Actinidia chinensis*, *Cornus florida*, *Rhododendron vialii*) were retrieved from the NCBI GenBank database (http://www.ncbi.nlm.nih.gov/, accessed on 8 July 2025) (Table 1). CodonW (version 1.4.2) (https://codonw.sourceforge.net/, accessed on 8 July 2025) was employed to calculate ENC, the overall GC content of each gene (GC), the GC content at the 3rd position of synonymous codons (GC3s), as well as the proportions of C, A, T, and G at the 3rd position of synonymous codon (C3s, A3s, T3s, G3s). The CAIcal server [85] was used to calculate the overall nucleotide composition (A, G, T, C), the total AT and AT3 contents, as well as the GC content at the first and second codon positions, denoted as GC1 and GC2, respectively.

### 4.2. Codon Adaptation Index (CAI) and Codon Bias Index (CBI)

The CAI value indicates how closely the usage frequency of synonymous codons in a coding region aligns with that of optimal codons. It ranges from 0 to 1, with higher values reflecting better adaptation and potentially higher levels of gene expression [86,87]. The CBI is another numerical measure used to quantify CUB. It measures the deviation between the observed frequency of a gene’s preferred codons and the expected frequency derived from the genome’s overall codon usage pattern [88]. Higher CBI values reflect a stronger bias toward the use of preferred codons. The CBI spans from 0 to 1, where a value of 1 means that only optimal codons are used, while a value below 0 suggests the absence of optimal codon usage [89].

### 4.3. Grand Average of Hydropathy (GRAVY) and Aromaticity (AROMA) Analysis

GRAVY and AROMA indices serve as quantitative measures to evaluate natural selection’s influence on codon usage, representing the relative abundances of hydrophobic and aromatic amino acids, respectively. Higher AROMA or GRAVY values indicate a greater proportion of aromatic or hydrophobic amino acids in the protein product [90]. These values were computed using CodonW.

### 4.4. Analyses of Relative Synonymous Codon Usage (RSCU) and Relative Synonymous Codon Usage Frequency (RFSC)

RSCU is a quantitative metric that represents the relative frequency of usage for each synonymous codon. An RSCU value exceeding 1 indicates that a codon is utilized more frequently than expected among synonymous codons for the same amino acid, reflecting a preferential or biased codon usage [91]. An RSCU value below 1 signifies that a codon is used less frequently than expected among synonymous codons for a given amino acid, indicating a reduced or negatively biased codon usage. An RSCU of 1 means that the codon is used with equal frequency or randomly in the RNA transcript, showing no bias toward a specific amino acid [91]. Synonymous codons with RSCU values above 1.6 are regarded as overrepresented, while those with values below 0.6 are considered underrepresented [92]. The RSCU is computed using the formula developed by Sharp and Li [93], as shown below (1):

(1) $$RSCU=\frac{X_{ij}}{\sum_{j}^{n_{i}}X_{ij}}n_{i}$$

In this formula, *X_ij_* indicates the number of times the *j*-th codon is used to encode the *i*-th amino acid, while *n_i_* denotes the number of synonymous codons that correspond to the *i*-th amino acid, ranging from 1 to 6 due to the redundancy of the genetic code.

RFSC represents the proportion of a specific codon’s usage relative to the total usage of all synonymous codons for a given amino acid. It is determined using the equation established by Sharp and Li [93], as outlined below (2):

(2) $$RFSC=\frac{X_{ij}}{\sum_{j}^{n_{i}}X_{ij}}$$

In this formula, *X_ij_* denotes the count of the *j*-th codon used to encode the *i*-th amino acid. Using this formula, the RFSC value is determined for each codon. A codon is classified as a high-frequency codon if it satisfies either of the following criteria: (1) the RFSC value of the codon is greater than 60% of the corresponding specific codon’s value. (2) the RFSC value of the codon exceeds the average frequency of all synonymous codons by more than 50% [23,29].

### 4.5. Optimal Codons Analysis

The effective number of codons (ENC) is a key measure used to evaluate how strongly codon usage is biased [26]. ENC values range from 20, which signifies maximum codon usage bias—where only a single synonymous codon is employed for each amino acid—to 61, indicating completely unbiased usage, with all synonymous codons utilized uniformly [94]. Lower ENC values correspond to a higher degree of codon usage bias [95]. The ENC values of the *PPO* genes were computed using CodonW. Based on these values, the genes were sorted in ascending order. The top 10% with the highest ENC values and the bottom 10% with the lowest were selected to represent low and high expression datasets, respectively. CodonW was then used to calculate the RSCU values for each of these datasets. Optimal codons were determined using the ΔRSCU method, where ΔRSCU represents the difference between the RSCU value in the high-expression dataset (RSCU_high_) and that in the low-expression dataset (RSCU_low_). A codon is regarded as optimal when its ΔRSCU is ≥0.08, with an RSCU value exceeding 1 in the high-expression dataset and falling below 1 in the low-expression dataset [26].

### 4.6. Comparative Analysis of Codon Utilization Frequency

Codon usage bias was evaluated by analyzing the frequency at which individual codons are utilized. The codon usage frequency of *CsPPO* genes and *PPO* genes from other plant species was analyzed using the CUSP tool available on the EMBOSS Explorer online platform (https://www.bioinformatics.nl/cgi-bin/emboss/cusp, accessed on 10 July 2025). Codon usage frequency data for *Escherichia coli*, *Saccharomyces cerevisiae*, *Arabidopsis thaliana*, *Triticum aestivum*, *Nicotiana tabacum*, and *Zea mays* were retrieved from the Codon Usage Database (https://dnahive.fda.gov/dna.cgi?cmd=codon_usage&id=537&mode=cocoputs, accessed on 10 July 2025). The codon usage frequencies of the *PPO* genes were compared with those of the referenced datasets. If the proportion is ≤0.5 or ≥2, it signifies a significant divergence in codon usage bias between the two organisms. Conversely, if the proportion falls within the range of 0.5 to 2, it indicates a highly similar codon usage preference, suggesting that the organism may be suitable as a host for heterologous gene expression [96].

### 4.7. ENC Plot Analysis

ENC plot analysis examines the correlation between ENC and GC3s within genes [44]. In this plot, GC3s values are represented on the x-axis, while ENC values are plotted on the y-axis for each gene. The expected ENC value was computed using the following Equation (3) [38]. When genes are located on or close to the expected ENC curve, it suggests that their CUB is primarily shaped by mutational pressure. Conversely, genes positioned significantly below the expected curve exhibit codon preferences shaped primarily by natural selection and additional evolutionary forces [44].

(3) $${ENC}_{exp}=2+GC3s+(\frac{29}{{GC3s}^{2}+{\left(1-GC3s\right)}^{2}})$$

### 4.8. Paritiy Rule 2 (PR2) Plot Analysis

PR2 plot analysis was employed to evaluate the relative contributions of natural selection and mutational pressure on nucleotide composition at the 3rd codon position. For each gene, the frequencies of the four nucleotides (T, A, G, and C) at the 3rd codon position were determined to calculate GC bias [G3/(G3 + C3)] and AT bias [A3/(A3 + T3)]. A PR2 bias plot was generated by plotting AT bias versus GC bias to illustrate the balance between purine and pyrimidine composition within genes. The center of the plot, with coordinates at (0.5, 0.5), denotes the equilibrium point where the frequencies of complementary bases are equal (A = T and G = C), indicating no bias. The position and orientation of each gene relative to the central point of the PR2 bias plot reflect the extent of deviation from parity rule 2 [97]. The aggregation of genes near the plot’s center indicates that codon usage bias is largely governed by mutational pressure, whereas substantial deviations from this central equilibrium suggest a stronger role of natural selection in shaping codon usage patterns [67,98].

### 4.9. Neutrality Analysis

Neutrality plot analysis involves constructing a regression line by plotting GC3s against GC12s. This method is employed to evaluate the respective influences of mutational pressure and natural selection in determining CUB within genes [99]. A regression line with a slope nearing 1 suggests that CUB is largely influenced by mutational pressure, with a relatively minor role played by natural selection. In contrast, a slope approaching 0 reflects a dominant influence of selection pressure in determining codon usage patterns [100].

### 4.10. Correspondence Analysis (COA)

COA was utilized to explore the underlying factors that may contribute to CUB. To characterize the CUB of *PPO* genes, the analysis was performed using the RSCU values of 59 codons, excluding ATG, TGG, and the three stop codons. Scatter plots were constructed with Axis 1 and Axis 2 representing the horizontal and vertical coordinates, respectively. The codon usage patterns were inferred based on the spatial distribution of data points within the plot.

### 4.11. Gene Expression Level Analysis

To elucidate the association between CUB and gene expression, synonymous codon usage order (SCUO) analysis was conducted. SCUO values for each gene were calculated using an online tool (https://www.genscript.com/tools/rare-codon-analysis, accessed on 10 July 2025) [101]. The calculation formula of SCUO is as follows (4) [102]:

(4) $$SCUO=\sum_{i=1}^{ni}\left(\frac{\sum_{j=1}^{ni}xij}{\sum_{i=1}^{18}\sum_{j=1}^{ni}xij}\right){SCUO}_{i}$$

where *j* is the codon for the *i*-th amino acid. The SCUO metric quantifies the bias in synonymous codon usage across the entire sequence, with values ranging from 0 to 1. Higher SCUO scores indicate stronger codon selection pressure and are typically correlated with elevated gene expression levels, reflecting adaptation for increased translational efficiency [103]. The measure independent of length and composition (MILC) is an index of gene expression level that quantifies the deviation in codon usage between a given gene and an expected codon distribution [104]. MILC values for each gene were computed using an online tool (https://www.genscript.com/tools/rare-codon-analysis, accessed on 10 July 2025) [101]. The formula to calculate MILC is (5) [104]:

(5) $$MILC=\frac{\sum_{a}M_{a}}{L}-C$$

Here, *L* denotes the gene length in codons, ensuring that the expected increase with a larger number of codons is considered. The correction factor *C* is applied to prevent overestimation of overall bias in relatively short sequences. *M_a_* represents the contribution of a given amino acid *a* to codon usage bias. The MILC scale ranges from 0, indicating low expression, to 1, indicating high expression [29].

### 4.12. Statistical Analysis

CodonW (version 1.4.2) (https://codonw.sourceforge.net/, accessed on 8 July 2025) was used to analyze various CUB metrics, such as the CAI and CBI. Additionally, CodonW was utilized to compute amino acid frequencies and conduct correspondence analysis. OriginPro 9.0 software was employed to carry out the statistical analyses. Spearman’s rank correlation test was utilized to evaluate the associations between variables, considering *p*-values below 0.05 as indicative of statistical significance.

## 5. Conclusions

This study provides the first comprehensive analysis of CUB in *CsPPO* genes. Analysis of base composition and RSCU revealed that *CsPPO* genes exhibit a preference for codons ending in G or C. A total of eight optimal codons were identified in the *CsPPO* genes, offering valuable insights for the optimization of gene expression. Comparative analyses with other *Camellia* and non-*Camellia* species confirmed both conserved and species-specific codon usage patterns. Evolutionary force analyses, including ENC, PR2, and neutrality plots, demonstrated that natural selection rather than mutational pressure is the predominant factor shaping CUB in *CsPPO* genes. Codon usage comparisons indicated that *Zea mays* is the most suitable host for their heterologous expression. These findings will not only expand the available *CsPPO* gene resources but also deepen our understanding of CUB in *CsPPO* genes, providing a solid theoretical foundation for future studies on their genetic and evolutionary dynamics.

## Figures and Tables

**Table 1:** Nucleotide compositional analysis of *PPO* genes.

Species	Accession No.	T3s%	C3s%	A3s%	G3s%	GC%	AT%	GC3s%	AT3%	GC1%	GC2%
*C. sinensis*	DQ513313.1	29.86	34.76	28.15	35.22	49.17	50.83	53.78	45.00	52.50	40.00
*C. sinensis*	MK977642.1	29.94	36.09	29.67	32.21	49.25	50.75	52.59	45.86	51.90	41.72
*C. sinensis*	MK977643.1	30.67	34.15	28.60	34.57	48.89	51.11	52.75	46.00	52.33	40.33
*C. sinensis*	MK977644.1	35.37	31.91	27.00	32.25	48.83	51.17	49.74	48.66	53.19	41.95
*C. sinensis*	MK977645.1	45.22	12.17	26.05	48.15	45.39	54.61	44.30	55.26	59.21	32.24
*C. sinensis*	MZ442717.1	30.67	34.15	28.38	34.73	49.06	50.94	52.92	45.83	52.50	40.50
*C. sinensis*	MZ442718.1	30.67	34.15	28.67	34.57	48.89	51.11	52.75	46.00	52.33	40.33
*C. sinensis*	FJ656220.1	30.41	34.49	28.44	34.57	49.06	50.94	53.09	45.67	52.33	40.50
*C. sinensis*	EU787433.1	30.67	34.15	28.67	34.57	48.89	51.11	52.75	46.00	52.33	40.33
*C. sinensis*	JX465712.1	30.67	34.15	28.67	34.57	48.89	51.11	52.75	46.00	52.33	40.33
*C. sinensis*	GQ214317.1	30.67	34.15	28.44	34.81	48.94	51.06	52.92	45.83	52.33	40.33
*C. sinensis*	AY659975.1	30.4	34.8	29.74	31.95	48.90	51.10	51.79	46.88	52.08	41.49
*C. sinensis*	FJ210643.1	30.12	34.84	28.51	34.65	49.06	50.94	53.36	45.33	52.00	40.50
*C. sinensis*	EF650017.1	30.88	34.15	28.51	34.65	48.89	51.11	52.75	46.00	52.33	40.33
*C. sinensis*	EF650016.1	30.53	34.02	28.15	35.14	49.25	50.75	53.18	45.58	52.75	40.57
*C. sinensis*	EF635860.1	30.67	34.15	28.67	34.57	48.94	51.06	52.75	46.00	52.50	40.33
*C. sinensis*	EF623826.1	30.67	34.15	28.6	34.57	48.89	51.11	52.75	46.00	52.33	40.33
*C. sinensis*	MH250121.1	30.67	34.15	28.67	34.57	48.83	51.17	52.75	46.00	52.33	40.17
*C. sinensis*	MH250120.1	30.66	34.36	28.08	34.98	49.11	50.89	53.18	45.50	52.83	40.00
*C. sinensis*	MH250119.1	29.8	34.49	28.08	35.47	49.33	50.67	53.78	45.00	52.50	40.50
*C. sinensis*	MH250118.1	30.25	34.57	28.31	35.14	49.22	50.78	53.44	45.33	52.67	40.33
*C. sinensis*	GQ129142.1	29.86	34.76	28.15	35.22	49.11	50.89	53.78	45.00	52.33	40.00
*C. sinensis*	MZ442720.1	30.82	33.88	28.83	34.48	48.83	51.17	52.49	46.33	52.33	40.50
*C. sinensis*	MZ442719.1	29.86	34.56	28.15	35.38	49.17	50.83	53.78	45.00	52.50	40.00
*C. lanceoleosa*	KAI7999995.1	37.45	29.76	27.27	31.68	48.38	51.62	47.66	50.67	53.68	42.14
*C. nitidissima*	ACM43505.1	31.30	26.93	32.00	48.60	48.60	51.40	49.04	49.33	53.69	41.44
*C. ptilophylla*	ABF19601.1	35.05	28.21	34.81	49.50	49.50	50.50	53.81	44.97	52.85	40.60
*A. chinensis*	PSR98570.1	32.26	22.14	41.00	50.75	50.75	49.25	55.77	42.93	53.41	41.76
*C. florida*	XM_059815174.1	28.63	27.19	31.03	47.60	47.60	52.40	46.00	52.74	52.57	42.95
*R. vialii*	XM_058340762.1	38.34	25.81	32.76	49.83	49.83	50.17	55.61	43.00	50.33	42.17

**Table 2:** Indices of codon usage bias for *PPO* genes.

Species	Accession No.	ENC	CBI	CAI	GRAVY	AROMA
*C. sinensis*	DQ513313.1	57.07	−0.048	0.196	−0.479466	0.09182
*C. sinensis*	MK977642.1	55.87	−0.064	0.196	−0.467012	0.091537
*C. sinensis*	MK977643.1	56.81	−0.056	0.197	−0.476628	0.09182
*C. sinensis*	MK977644.1	57.73	−0.092	0.193	−0.411092	0.092437
*C. sinensis*	MK977645.1	49.01	−0.239	0.161	−0.318543	0.046358
*C. sinensis*	MZ442717.1	56.83	−0.054	0.197	−0.489149	0.09015
*C. sinensis*	MZ442718.1	56.89	−0.056	0.196	−0.489983	0.09182
*C. sinensis*	FJ656220.1	56.63	−0.05	0.197	−0.492321	0.09182
*C. sinensis*	EU787433.1	56.89	−0.056	0.196	−0.489983	0.09182
*C. sinensis*	JX465712.1	56.89	−0.056	0.196	−0.489983	0.09182
*C. sinensis*	GQ214317.1	56.86	−0.054	0.197	−0.489983	0.09182
*C. sinensis*	AY659975.1	55.80	−0.015	0.2	−0.451826	0.093913
*C. sinensis*	FJ210643.1	56.66	−0.047	0.198	−0.492321	0.093489
*C. sinensis*	EF650017.1	56.64	−0.059	0.195	−0.479633	0.09182
*C. sinensis*	EF650016.1	56.37	−0.044	0.199	−0.511539	0.091973
*C. sinensis*	EF635860.1	56.97	−0.056	0.196	−0.493155	0.09015
*C. sinensis*	EF623826.1	56.81	−0.056	0.197	−0.476628	0.09182
*C. sinensis*	MH250121.1	56.79	−0.055	0.196	−0.486644	0.09182
*C. sinensis*	MH250120.1	56.19	−0.041	0.201	−0.482304	0.09182
*C. sinensis*	MH250119.1	55.32	−0.039	0.198	−0.478798	0.093489
*C. sinensis*	MH250118.1	55.52	−0.033	0.204	−0.53005	0.09182
*C. sinensis*	GQ129142.1	56.93	−0.046	0.198	−0.47813	0.09182
*C. sinensis*	MZ442720.1	57.47	−0.05	0.198	−0.489316	0.09182
*C. sinensis*	MZ442719.1	57.11	−0.045	0.197	−0.485309	0.09182
*C. lanceoleosa*	KAI7999995.1	57.76	−0.105	0.192	−0.404523	0.092127
*C. nitidissima*	ACM43505.1	57.41	−0.099	0.189	−0.395798	0.090756
*C. ptilophylla*	ABF19601.1	55.74	−0.035	0.197	−0.493109	0.092437
*A. chinensis*	PSR98570.1	56.23	−0.101	0.185	−0.491167	0.093333
*C. florida*	XM_059815174.1	55.09	−0.025	0.214	−0.528904	0.094684
*R. vialii*	XM_058340762.1	55.00	−0.026	0.196	−0.46995	0.085142

**Table 3:** High-frequency (HF) codons identified in *PPO* genes of *C. sinensis* and other plant species.

Species	Number of HF Codons	HF Codons Identified
*C. sinensis*	18	TTC, CTC, ATT, GTG, TCC, CCG, ACC, GCC, TAC, CAT, CAA, AAC, AAG, GAT, GAG, TGC, CGA, GGG
*C. lanceoleosa*	20	TTC, CTT, ATT, GTG, TCC, CCT, ACC, GCC, TAT, TAC, CAT, CAA, AAG, GAT, GAG, TGT, CGA, AGA, GGG
*C. nitidissima*	21	TTC, TTG, ATT, GTG, TCC, CCT, CCC, ACC, GCT, TAT, TAC, CAT, CAA, AAT, AAG, GAT, GAG, TGT, TGC, CGG, GGG
*C. ptilophylla*	19	TTC, CTC, ATT, GTG, TCC, CCG, ACC, GCC, TAC, CAT, CAA, AAC, AAG, GAT, GAG, TGC, CGA, GGG
*A. chinensis*	17	TTT, TTG, ATT, GTG, TCC, CCG, ACC, GCC, TAT, CAC, CAA, AAT, AAG, GAT, GAG, AGG, GGG
*C. florida*	20	TTT, TTG, ATA, GTG, TCC, CCA, ACC, GCT, TAC, CAT, CAA, AAT, AAG, GAT, GAG, TGT, TGC, AGG, GGT
*R. vialii*	20	TTC, CTT, ATC, GTG, TCC, CCC, ACC, GCC, TAC, CAT, CAA, AAC, AAA, AAG, GAT, GAG, TGC, AGA, AGG, GGG

**Table 4:** SCUO and MILC analyses of *PPO* genes in *C. sinensis* and other plant species.

Species	SCUO	MILC
*C. sinensis*	0.06	0.50
*C. lanceoleosa*	0.06	0.50
*C. nitidissima*	0.06	0.50
*C. ptilophylla*	0.06	0.49
*A. chinensis*	0.07	0.49
*C. florida*	0.07	0.48
*R. vialii*	0.06	0.49

**Table 5:** Correlation analysis between ENC values, CAI values and codon composition of *CsPPO* genes.

Indices	GC	GC1	GC2	GC3s	A3s	T3s	C3s	G3s	AT3
ENC									
r	0.87924 *	−0.90933 *	0.8891 *	0.79626 *	0.55742 *	−0.82919 *	0.90027 *	−0.88771 *	−0.82775 *
*p*	<0.05	<0.05	<0.05	<0.05	<0.05	<0.05	<0.05	<0.05	<0.05
CAI									
r	−0.4184 *	0.47938 *	−0.16986	−0.68726 *	−0.5215 *	0.64212 *	−0.47633 *	0.18885	0.64205 *
*p*	<0.05	<0.05	<0.05	<0.05	<0.05	<0.05	<0.05	0.37683	<0.05

* *p*-value < 0.05.

**Table 6:** Correlation analysis among ENC, A3s, T3s, G3s, C3s, GC3s, GRAVY and AROMA values.

Indices	ENC	A3s	T3s	C3s	G3s	GC3s
GRAVY						
r	−0.74322 *	−0.64531 *	0.91663 *	−0.86921 *	0.68189 *	−0.93131 *
*p*	<0.05	<0.05	<0.05	<0.05	<0.05	<0.05
AROMA						
r	0.92501 *	0.68872 *	−0.93759 *	0.98578 *	0.68189 *	0.89588 *
*p*	<0.05	<0.05	<0.05	<0.05	<0.05	<0.05

* *p*-value < 0.05.

## Data Availability

The data presented in this study are available on request from the corresponding author.

## Supplementary Materials

The following supporting information can be downloaded at: https://www.mdpi.com/article/10.3390/plants14193074/s1, Table S1. Nucleotide compositional analysis of *PPO* genes. Table S2. RSCU and RFSC values of the codons in *PPO* genes. Table S3. The RSCU values and ΔRSCU value in the high and low expression library of *PPO* genes. Table S4. Comparison of codon usage frequency between *PPO* genes and six organisms. Table S5. Analysis of amino acid usage frequency in *PPO* genes of *C. sinensis* and other plant species. Table S6. Correlation between SCUO and MILC in PPO genes of *C. sinensis* and other plant species. Table S7. Correlation analysis between ENC values, CAI values, AROMA values, GRAVY values and codon composition of *PPO* genes of other *Camellia* species and non-*Camellia* species.
